# Characteristics of the resistome and the potential for bloodstream infections in patients with gut colonization by *Klebsiella pneumoniae* undergoing hematopoietic stem cell transplantation

**DOI:** 10.3389/fcimb.2026.1727300

**Published:** 2026-03-27

**Authors:** Jiacheng Qiu, Zhenxin Zhuang, Rongping Zhu, Yingping Cao, Xiaohong Xu

**Affiliations:** 1 Department of Laboratory Medicine, Fujian Medical University Union Hospital, Fuzhou, Fujian, China; 2 Department of Laboratory Medicine, Jinjiang Hospital of Traditional Chinese Medicine Affiliated to Fujian University of Traditional Chinese Medicine, Quanzhou, Fujian, China; 3 Department of Blood Transfusion, Fuzhou University Affiliated Provincial Hospital, Fuzhou, Fujian, China; 4 Fujian Medical University Union Clinical College, Fuzhou, Fujian, China

**Keywords:** bloodstream infections, colonization, hematopoietic stem cell transplantation, *Klebsiella pneumoniae*, non-antibacterial drugs, risk factors

## Abstract

**Background:**

Hematopoietic stem cell transplantation (HSCT) patients are at high risk for intestinal colonization by *Klebsiella pneumoniae* (Kp), potentially leading to Kp-associated bloodstream infections (BSI). This study aims to determine the incidence of Kp colonization, the risk of its progression to Kp-BSI, and the associated risk factors in HSCT patients.

**Methods:**

Between August 2022 and December 2023, perianal swab specimens were prospectively collected from HSCT recipients. Bacterial isolates were identified using matrix-assisted laser desorption/ionization time-of-flight mass spectrometry (MALDI-TOF/MS). Polymerase chain reaction (PCR) was employed to screen for prevalent antimicrobial resistance genes. The minimum inhibitory concentration (MIC) of colonizing strains to common antimicrobial agents was determined using the VITEK 2 automated system (bioMérieux, France). Risk factors associated with Kp colonization and subsequent BSI were analyzed by logistic regression.

**Results:**

Among 409 HSCT recipients, 112 (27.4%) demonstrated pre-transplant Kp colonization, including 14 cases of carbapenem-resistant Kp (CRKp). Subsequent Kp-BSI occurred in 14 colonized patients. The colonizing strains exhibited the highest susceptibility rates to carbapenems among all antimicrobial classes tested. Multivariate analysis identified the following independent risk factors for Kp colonization: higher Hematopoietic Cell Transplantation Comorbidity Index (HCT-CI), fever, and use of posaconazole, acyclovir, and proton pump inhibitors (omeprazole). Colonized patients had a significantly higher risk of developing Kp-BSI within 100 days post-HSCT (*P* < 0.0001).

**Conclusions:**

Kp colonization significantly increases the risk of subsequent BSI in HSCT patients. Studies have found that rational management of non-antibacterial drugs (such as strictly evaluating the indications for proton pump inhibitors) can reduce the incidence of Kp colonization. Our data suggest that it is necessary to enhance awareness of the risks associated with bacterial colonization before transplantation.

## Introduction

Hematopoietic stem cell transplantation (HSCT) is an effective treatment strategy for hematological malignancies such as leukemia and lymphoma as well as primary immunodeficiency diseases, and it is of great significance for children, adolescents, and adults (Wilson et al., 2014; Yan et al., 2024). In 2023, a total of 14,952 cases of HSCT were completed in 216 medical institutions in China, ranking first in the world (Xu et al., 2024). HSCT plays a pivotal role in prolonging the survival of patients with hematological diseases. However, approximately 30%-50% of patients have an increased non-recurrent mortality rate due to infection complications, among which bloodstream infection (BSI) plays an important role: BSI incidence following HSCT can range from 25% to 40%, and drug-resistant bacterial infections can lead to mortality rates of 20% to 35% (Gudiol and Carratala, 2014).

More and more data suggest that gut dysbiosis is a primary mechanism that predisposes HSCT recipients to serious infections. Chemotherapy/radiation used in pre-transplant conditioning, along with broad-spectrum antibiotics and immunosuppressive drugs, damage the intestinal mucosal barrier, disrupt the ecological balance, and reduce commensal microbiota (Yu et al., 2020). This imbalance in the gut microbiome allows for the opportunistic growth of harmful, multidrug-resistant pathogens, especially *Enterobacteriaceae* (Shono and Van Den Brink, 2018). Among these, *Klebsiella pneumoniae* (Kp) represents a significant threat (Taur et al., 2014; Ni et al., 2024, 2022). Kp, a highly virulent and multidrug-resistant member of the *Enterobacteriaceae* family, has been reported by Peled et al. to potentially undergo significant expansion and become a dominant pathogen in HSCT patients when gut microbiota diversity is severely depleted (Peled et al., 2020). These strains can translocate across the compromised intestinal epithelial barrier, triggering lethal BSIs. Moreover, due to limited therapeutic options for carbapenem-resistant *Klebsiella pneumoniae* (CRKp), the 30-day mortality rate in affected patients exceeded 50% (De Souza et al., 2024). Consequently, Kp colonization and infection profoundly impact the clinical outcomes of highly immunocompromised populations, including intensive care unit (ICU) patients and solid organ transplant (SOT) or HSCT recipients (Diekema et al., 2019; Zhang et al., 2024; Fan et al., 2025). CRKp colonization is notably prevalent in ICUs, with reported rates ranging from 15.2% to 49% (Qin et al., 2020). The risk of CRKp colonization is heightened by gut microbiota dysbiosis common in HSCT patients. This dysbiosis is characterized by a reduced abundance and diversity of commensal anaerobic bacteria that provide colonization resistance against pathogens. The loss of these potential antagonistic bacteria creates an ecological niche that facilitates the expansion of resistant *Enterobacteriaceae* like CRKp (Hu et al., 2025). In HSCT recipients specifically, rectal CRKp colonization has been established as an independent risk factor for subsequent carbapenem-resistant *Enterobacterales* (CRE) infections, particularly CRE-associated BSIs (Cao et al., 2022). Furthermore, Kp is frequently identified as the predominant colonizing bacterium in the pharynx of HSCT patients (Ge et al., 2022). These findings collectively underscore the multi-site colonization patterns of Kp in susceptible populations and their associated clinical risks. However, current evidence is largely limited to single-center or retrospective analyses, lacking integrated investigation into the dynamic carriage patterns of resistance genes and associated clinical risk factors.

Based on this gap, this study aims to elucidate the resistome characteristics of gut-colonizing Kp strains in HSCT patients and their causal relationship with BSI through molecular epidemiological investigation combined with longitudinal clinical data tracking.

## Materials and methods

### Study design and population

We conducted a single-center, prospective observational case-control study at the Hematology Department of Fujian Medical University Union Hospital. The study involved active screening for Kp colonization in all patients undergoing their first HSCT between August 1, 2022, and December 31, 2023. The study included patients receiving umbilical cord blood, bone marrow, or peripheral blood stem cell transplants. The screening method involved collecting perianal swabs (once upon admission and twice weekly after entering the transplant unit).

Cases were defined as patients who had at least one perianal swab test positive for Kp prior to HSCT. Each patient was included only once, at the time of the first isolation of Kp from a perianal swab (index culture), even if multiple Kp colonizations were reported. The control group consisted of contemporaneous patients who did not develop Kp colonization. Informed consent was obtained from all participants.

### Study cohort and enrollment

This study was conducted at a large first-class tertiary hospital in China, with an annual cumulative admission rate of approximately 180,000 patients. Among them, the Hematology Department of Fujian Medical University Union Hospital pioneered HSCT in 1989, making it one of the earliest medical institutions in China to perform stem cell transplantation.

Patient Inclusion Criteria: (i) Admitted to the Bone Marrow Transplantation Center of the Hematology Department; (ii) Scheduled for HSCT; (iii) Kp detected in perianal swabs during hospitalization. Exclusion Criteria: (i) Patients with incomplete medical records; (ii) Patients with missing bacterial strains; (iii) Patients with detected colonization by bacteria other than Kp.

### Data sources and variables

Data were collected using standardized case report forms. Underlying diseases were documented according to the Eastern Cooperative Oncology Group (ECOG) performance status score (Jung et al., 2025). The clinical data of patients were described, including demographic characteristics, disease and treatment characteristics, transplant complications, outcomes. The following variables were assessed: sex, age at transplantation, diagnosis, donor relationship, stem cell source, conditioning intensity. Established clinical criteria were used to diagnose: Graft-versus-Host Disease (GVHD) (Glucksberg et al., 1974), engraftment syndrome in HSCT recipients (Smith et al., 2011). Chemotherapy and/or radiotherapy were defined as the administration of cytotoxic anti-neoplastic agents or ionizing radiation for curative or palliative cancer treatment. The Hematopoietic Cell Transplantation Comorbidity Index (HCT-CI) was utilized as a tool to assess the risk of mortality following hematopoietic cell transplantation. This index summarizes weighted scores for pre-transplant individual organ dysfunctions; scores range from 0 to a theoretical maximum of 26, with higher scores indicating a greater risk of post-transplant mortality (Sorror et al., 2005). Patients were followed until either hospital discharge or in-hospital death.

### Outcome definitions

Kp colonization carriers were defined as patients from whom Kp was isolated from perirectal swabs in the absence of signs and symptoms of invasive infection. Kp-BSI was defined as a BSI documented by a positive blood culture (BC) for a Kp strain (at least one sample) and clinical signs of systemic inflammatory response syndrome (SIRS) (Zarkotou et al., 2011). The BSI episode was considered as the date of index BC collection (i.e. the first BC yielding the study isolate).

### Microbiological study

Bacterial isolates were obtained from perianal swabs inoculated onto Mueller-Hinton Agar (Antu Bio, China). Isolates were identified using matrix-assisted laser desorption/ionization time-of-flight mass spectrometry (MALDI-TOF MS; Bruker Daltonics). Carbapenem resistance (meropenem or imipenem) was confirmed by disk diffusion testing, defining CRE as Enterobacteriaceae resistant to either carbapenem (Castagnola et al., 2019). For CRKp isolates, carbapenemase production was assessed by modified Hodge test (Tamma and Simner, 2018). Polymerase chain reaction (PCR) and sequencing were used to detect antimicrobial resistance genes (ARGs) (Kiaei et al., 2019). Sequence results were analyzed using BLAST (http://www.ncbi.nlm.nih.gov/BLAST). The primers used for this analysis can be found in **Supplementary Table 1**.

### Determination of minimum inhibitory concentrations

The MICs of the colonizing *Klebsiella pneumoniae* isolates against a panel of antimicrobial agents were determined using VITEK 2 automated system (bioMérieux, Marcy-l’Étoile, France). Briefly, pure colonies were suspended in 0.45% saline to achieve a turbidity equivalent to a 0.5 McFarland standard. The suspension was then loaded into the appropriate VITEK 2 AST-GN13 test card according to the manufacturer’s instructions. The cards were incubated and read automatically by the instrument, which reported the MIC value for each antimicrobial agent. *Escherichia coli* ATCC 25922 and *Pseudomonas aeruginosa* ATCC 27853 were used as quality control strains for each batch of testing. The interpretation of the results was based on the 2023 Clinical and Laboratory Standards Institute (CLSI 2023) guidelines.

### Statistical analysis

Data were analyzed using IBM SPSS ver. 21.0 statistical software (IBM Co., Armonk, NY, USA). Frequency tables (n, %) for categorical variables and descriptive statistics (mean, median, standard deviation) for numerical variables were used. Comparisons of categorical variables were analyzed by the Chi square test. Logistic regression (Backward LR) methods (univariate, multivariate) were used to determine the risk factors for Kp colonization and BSI. Statistical significance was assigned to a *P* value of less than 0.05.

## Results

### Patient population

**Figure 1:**
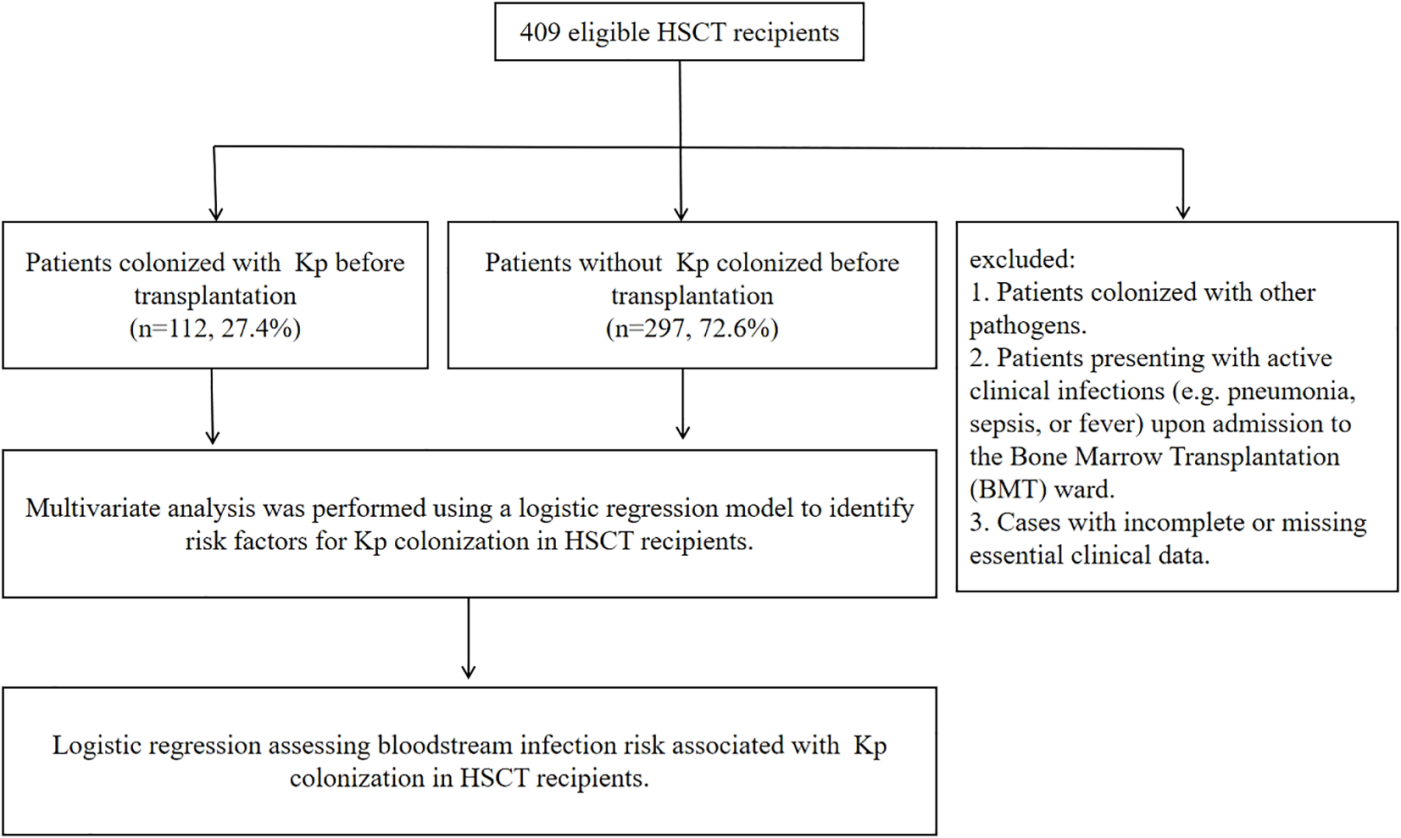
Patients’ enrollment and exclusion of our study.

During the study period (August 1, 2022, to December 31, 2023), 3,041 perianal swab specimens were collected from 416 HSCT candidates. Kp was isolated from 210 specimens (screening positivity rate: 6.9%, 210/3,041), corresponding to 119 colonized patients (colonization rate: 28.6%, 119/416). Among 119 unique patient-derived Kp isolates (excluding duplicate specimens from the same patient/timepoint), 7 strains failed revival due to improper storage. The process for screening patients is shown in **Figure 1**. Consequently, 112 viable isolates underwent antimicrobial susceptibility testing by disk diffusion for imipenem, meropenem, and ertapene. CRKp colonization was identified in 14 patients (3.4%, 14/409) based on phenotypic resistance.

The final cohort comprised 409 HSCT recipients. Demographic and clinical characteristics included: 224 males (54.8%) and 185 females (45.2%), with a mean age of 38.8 ± 17.9 years (range: 1–76 years). The mean hospital stay was 34.6 ± 20.5 days. Predominant diagnoses were acute myeloid leukemia (26.65%), multiple myeloma (21.3%), and acute lymphoblastic leukemia (20.8%) (**Table 1**).

**Table 1:** Clinical characteristics and transplant data of Kp-colonization group and non-colonization group.

Characteristics	Kp-colonization group (n = 112)	Non-colonization group (n = 297)
Age, years, median(IQR)	43.5 (1,76)	40 (1,68)
Sex, *n* (%)		
Male	66 (58.9%)	158 (53.2%)
Female	46 (41.1%)	139 (46.8%)
HCT-CI		
≥3	54 (48.2%)	30 (10.1%)
<3	58 (51.8%)	267 (89.9%)
Underlying disease, *n* (%)		
AML	33 (29.5%)	76 (25.6%)
ALL	29 (25.9%)	56 (18.9%)
MM	25 (22.3%)	62 (20.9%)
LYM	11 (9.8%)	35 (11.8%)
MDS	9 (8.0%)	24 (8.1%)
AA	1 (0.9%)	25 (8.4%)
Others	4 (3.6%)	19 (6.4%)
Transplant complications		
Engraftment syndrome	24 (21.4%)	16 (5.4%)
HLH	2 (1.8%)	1 (0.3%)
GVHD	2 (1.8%)	0
Donor (%)		
Autologous	41 (36.6%)	123 (41.4%)
Haploidentical	24 (21.4%)	76 (25.6%)
MSD	47 (42.0%)	98 (33.0%)
MUD	0	0
Conditioning regimen (%)		
MAC	95 (84.8%)	265 (89.2%)
RIC or NMA	17 (15.2%)	32 (10.8%)
Graft source (%)		
BM	7 (6.3%)	39 (13.1%)
UCB	33 (29.5%)	40 (13.5%)
PB (only auto-HSCT)	41 (36.6%)	123 (41.4%)
BM+UCB	31 (27.7%)	94 (31.6%)
BM+PB	0	1 (0.3%)
ABO incompatibility (%)		
Compatible	67 (59.8%)	180 (60.6%)
Minor mismatch	12 (10.7%)	24 (8.1%)
Major/bidirectional mismatch	17 (15.2%)	38 (12.8%)
half blood type matched in combined transplantation	11 (9.8%)	46 (15.5%)
ABO-incompatible in combined transplant	5 (4.5%)	9 (3.0%)
Donor-recipient gender match (%)		
Female to male	11 (9.8%)	17 (5.7%)
Male to female	14 (12.5%)	34 (11.4%)
Others	87 (77.7%)	246 (82.8%)

Kp, *Klebsiella pneumoniae*; IQR, interquartile range; HCT-CI, hematopoietic cell transplantation–comorbidity index; AML, acute myelogenous leukemia; ALL, acute lymphoblastic leukemia; MM, multiple myeloma; LYM, lymphoma; MDS, myelodysplastic; AA, aplastic anemia; HLH, hemophagocytic lymphohistiocytosis; GVHD, graft-versus-host disease; MSD, matched sibling donor; MUD, matched unrelated donor; MAC, myeloablative conditioning; RIC, reduced intensity conditioning; NMA, non-myeloablative conditioning; BM, bone marrow; UCB, umbilical cord blood; PB, peripheral blood.

### Antimicrobial susceptibility of colonizing *Klebsiella pneumoniae* isolates

A total of 112 unique colonizing Kp isolates were tested against 15 antimicrobial agents. The detailed MIC distribution and susceptibility profile are summarized in the **Table 2**. The colonizing strains exhibited the highest susceptibility rates to carbapenems (imipenem, meropenem, and ertapenem) among all antimicrobial classes tested. Amikacin also retained good potency, exhibiting a susceptibility rate of 80.36%. Among cephalosporins, susceptibility varied widely: Cefotetan demonstrated the highest activity (89.29% susceptible), followed by cefepime (66.96%) and ceftazidime (52.68%). In contrast, susceptibility to ceftriaxone and cefazolin was substantially lower (36.61% and 29.46%, respectively). This pattern is consistent with a high prevalence of ESBL-positive phenotypes (51.79%).

**Table 2:** MIC distribution and susceptibility profile of 112 *Klebsiella pneumoniae* isolates.

Antimicrobial Agent	MIC Range (µg/mL)	Susceptible (n)	Intermediate (n)	Resistant (n)	%S (n)
Cephalosporins					
Cefazolin	≤2 – ≥64	33	0	79	29.46
Cefotetan	≤4 – ≥64	100	1	11	89.29
Ceftriaxone	≤1 – ≥64	41	0	71	36.61
Cefepime	≤1 – ≥64	75	5	32	66.96
Ceftazidime	≤1 – ≥64	59	6	47	52.68
Carbapenems					
Imipenem	≤1 – ≥16	99	3	10	88.39
Meropenem	≤0.5 – ≥8	111	0	1	99.11
Ertapenem	≤0.5 – ≥8	109	0	3	97.32
Monobactams					
Aztreonam	≤1 – ≥64	53	1	58	47.32
Aminoglycosides					
Gentamicin	≤1 – ≥16	39	3	70	34.82
Tobramycin	≤1 – ≥16	41	25	46	36.61
Amikacin	≤2 – ≥64	90	0	22	80.36
Fluoroquinolones					
Ciprofloxacin	≤0.25 – ≥4	25	20	67	22.32
Levofloxacin	≤0.25 – ≥8	19	0	93	16.96
Nitrofurans					
Nitrofurantoin	≤16 – ≥512	19	57	36	16.96

### Detection of antimicrobial resistance genes

Among 112 non-duplicate Kp isolates, extended-spectrum β-lactamase (ESBL) genes were detected as follows: *SHV* (85.7%, 96/112) predominated, followed by *TEM* (53.6%, 60/112), *CTX-M-1* (50.0%, 56/112), *CTX-M-10* (49.1%, 55/112), *CTX-M-9* (12.5%, 14/112), and *CTX-M-14* (12.5%, 14/112). No *CTX-M-2* or *CTX-M-8* variants were identified. Co-carriage analysis revealed 65 isolates (58.0%) harbored ≥1 *CTX-M* variant (*CTX-M-1*/*9*/*10*/*14*), while 50 (44.6%) carried both *SHV* and *TEM*, and 41 (36.6%) possessed all 6 ESBL genes. Quinolone resistance genes were distributed as: *gyrA* (96.4%, 108/112), *qnrS* (62.5%, 70/112), *qnrB* (32.1%, 36/112), and *qnrA* (7.1%, 8/112). *qepA* was undetected. Aminoglycoside resistance genes included: *aac (3)-II* (54.5%, 61/112), *ant (3’’)-I* (48.2%, 54/112), *aac (6’)-Ib* (46.4%, 52/112), *rmtB* (17.0%, 19/112), and *armA* (5.4%, 6/112). Carbapenemase genes were identified in 6 isolates (5.4%, 6/112): *bla_KPC_* (1.8%, 2/112) and *bla_NDM_* (3.6%, 4/112). No *IMI*, *GIM*, *SME*, *bla_IMP_*, *bla_VIM_*, *bla_OXA-181_*, or *bla_OXA-48_*variants were detected.

All colonizing strains carried ≥1 ESBL gene (100%), with 97.3% (109/112) harboring quinolone resistance determinants, 85.7% (96/112) aminoglycoside resistance genes, and 5.4% (6/112) carbapenemase genes. Co-occurrence of ESBL and quinolone resistance genes was observed in 93.8% (105/112), while 84.8% (95/112) carried both ESBL and aminoglycoside resistance genes.

**Figure 2:**
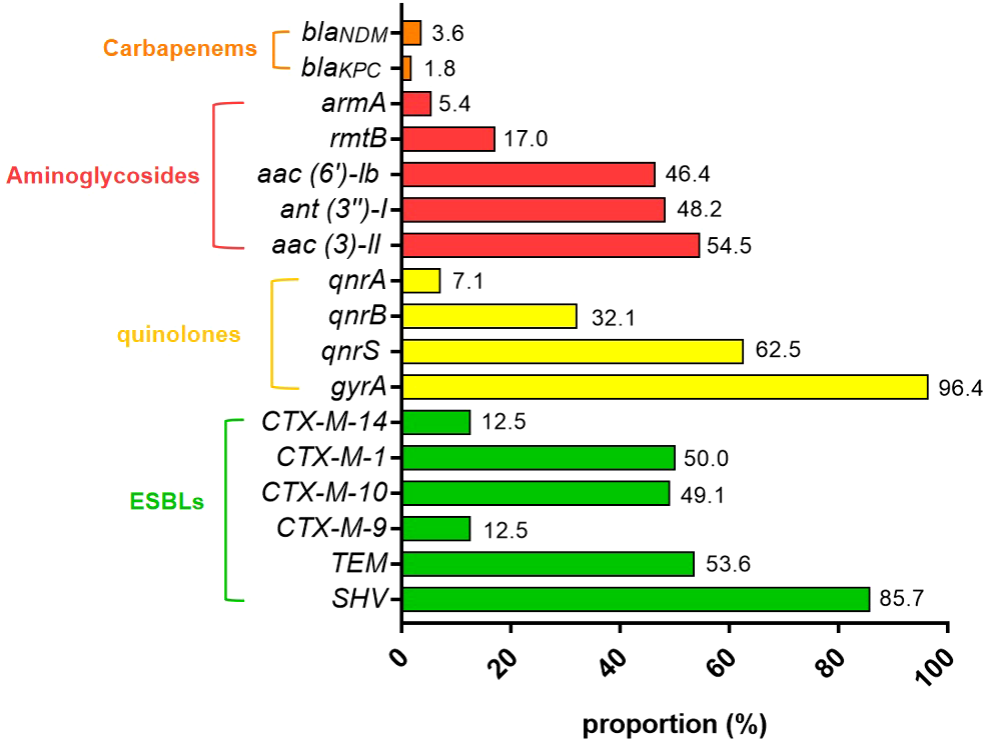
Molecular characterization of resistance genes in Kp colonization isolates as detected by PCR.

Among the 14 CRKp isolates, carbapenemase genes were detected in 6 strains: 2 *bla_KPC_* strains and 4 *bla_NDM_* strains, with 8 strains lacking identifiable carbapenemase genes (**Figure 2**).

### Risk factors for colonization

**Figure 3:**
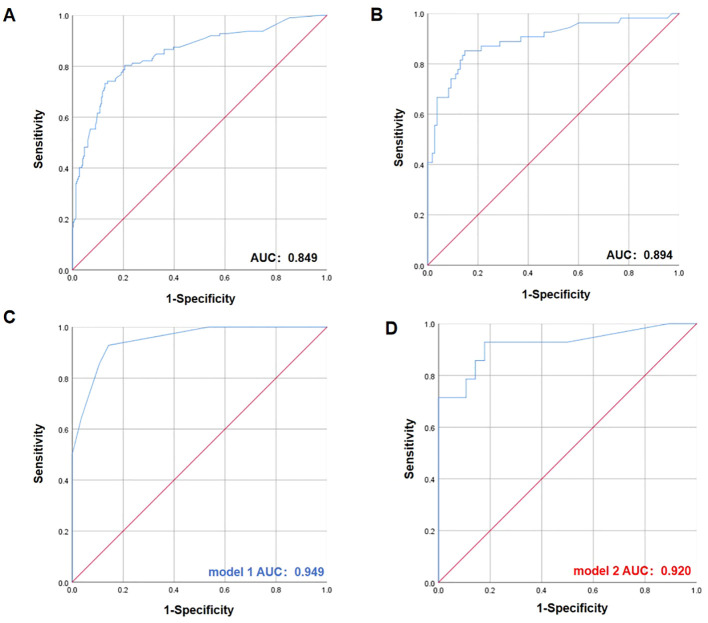
Receiver operator characteristic (ROC) curve analysis for the scoring system. **(A)** ROC curve for predicting clinical risk factors in Kp-colonized patients. **(B)** ROC curve for predicting clinical risk factors in CTX-M-Kp-colonized patients. **(C)** ROC curve of Model 1 for predicting CRKp colonization, incorporating non-antibiotic risk factors. **(D)** ROC curve of Model 2 for predicting CRKp colonization, focusing on prior antibiotic exposure.

In this case-control analysis (112 colonized *vs*. 297 non-colonized HSCT recipients), multivariate analysis identified several independent risk factors significantly associated with the colonization (*P* < 0.05): higher HCT-CI (OR 8.557, 95% CI 4.474-16.366; *P* < 0.0001), posaconazole exposure (OR 3.213, 95% CI 1.796-5.749; *P* < 0.01), acyclovir administration (OR 3.135, 95% CI 1.530-6.425; *P* < 0.0001), omeprazole use (OR 2.440, 95% CI 1.280-4.651; *P* < 0.05), febrile episodes (OR 2.142, 95% CI 1.167-3.391; *P* < 0.05) (**Table 3**). Based on the identified risk factors, we developed a risk scoring system to predict Kp colonization in HSCT patients. Each factor was assigned weighted points based on its risk contribution, resulting in a total score ranging from 0 to 6 points (**Table 4**). To evaluate the predictive performance of the model and determine the optimal diagnostic threshold, we performed receiver operating characteristic (ROC) curve analysis (**Figure 3A**). The ROC analysis confirmed that the model possesses high discriminatory power, with an area under the curve (AUC) of 0.849.

**Table 3:** Univariate analysis and multivariate logistic regression analysis of risk factors for Kp colonization in colonized versus non-colonized patients.

Variables	Kp colonized n (%)	Non-colonized n (%)	Bivariate		Multivariate	
**Total no. of patients**	**N=112 (27.4)**	**N=297 (72.6)**	**OR (95%CI)**	**p**	**OR (95%CI)**	**p**
**Age (years)**	41.3 ± 16.9	37.8 ± 18.3	1.011 (0.999-1.024)	0.081		
**Sex (male)**	66 (58.9%)	158 (53.2%)	0.792 (0.510-1.230)	0.300		
**HCT-CI (≥3)**	54 (48.2%)	30 (10.1%)	8.286 (4.882-14.063)	0.000	8.557 (4.474-16.366)	0.000
**ECOG (3 to 5)**	16 (14.3%)	40 (13.5%)	1.071 (0.573-2.001)	0.830		
**Hospital stays (>30 days)**	64 (57.1%)	146 (49.2%)	1.379 (0.890-2.137)	0.150		
**No. of admissions (>5 times)**	84 (75.0%)	220 (74.1%)	0.952 (0.577-1.571)	0.848		
**Underlying Disease**	**n (%)**	**n (%)**	**OR (95% CI)**	**p**	**OR (95% CI)**	**p**
AML	33 (29.5%)	76 (25.6%)	0.485 (0.153-1.536)	0.218		
ALL	29 (25.9%)	56 (18.9%)	0.407 (0.126-1.307)	0.131		
MM	25 (22.3%)	62 (20.9%)	0.522 (0.161-1.689)	0.278		
LYM	11 (9.8%)	35 (11.8%)	0.670 (0.187-2.393)	0.537		
MDS	9 (8.0%)	24 (8.1%)	0.561 (0.150-2.107)	0.392		
AA	1 (0.9%)	25 (8.4%)	5.263 (0.543-50.998)	0.152		
Others	4 (3.6%)	19 (6.4%)				
Comorbidity						
Hypertension	11 (9.8%)	22 (7.4%)	1.361 (0.637-2.908)	0.426		
Diabetes mellitus	7 (6.3%)	18 (6.1%)	1.033 (0.420-0.545)	0.943		
Solid tumor	2 (1.8%)	16 (5.4%)	0.319 (0.072-1.412)	0.132		
Cardiac disease	17 (15.2%)	23 (7.7%)	2.132 (1.092-4.162)	0.027	1.783 (0.731-4.347)	0.204
Respiratory disease	28 (25.0%)	53 (17.8%)	1.535 (0.912-2.583)	0.107		
Gastrointestinal disease	12 (10.7%)	23 (7.7%)	1.430 (0.686-2.980)	0.340		
Hepatic disease	25 (22.3%)	62 (20.9%)	1.089 (0.644-1.842)	0.750		
Urinary system disease	15 (13.4%)	41 (13.8%)	0.966 (0.511-1.824)	0.914		
**Invasive operation**	**n (%)**	**n (%)**	**OR (95% CI)**	**p**	**OR (95% CI)**	**p**
Past surgical history	32 (28.6%)	71 (23.9%)	1.273 (0.781-2.076)	0.333		
PICC	108 (96.4%)	279 (93.9%)	1.742 (0.576-5.264)	0.325		
Femoral vein catheter	4 (3.6%)	9 (3.0%)	1.185 (0.358-3.929)	0.781		
**Fever**	52 (46.4%)	57 (19.2%)	3.649 (2.280-5.841)	0.000	2.069 (1.097-3.902)	0.025
History of antibiotic intake within last 3 months						
β-lactam/inhibitor						
Piperacillin/tazobactam	29 (25.9%)	31 (10.4%)	2.998 (1.707-5.265)	0.000	1.889 (0.880-4.054)	0.103
Cefoperazone/sulbactam	48 (42.9%)	102 (34.3%)	1.434 (0.919-2.236)	0.112		
Cephalosporins						
Cefdinir	31 (27.7%)	64 (21.5%)	1.393 (0.847-2.292)	0.192		
Ceftazidime	6 (5.4%)	14 (4.7%)	1.144 (0.429-3.055)	0.788		
Aminoglycosides						
Gentamicin	96 (85.7%)	290 (97.6%)	0.145 (0.058-0.363)	0.000		
Amikacin	10 (8.9%)	7 (2.4%)	4.062 (1.506-10.951)	0.006	1.969 (0.487-7.956)	0.342
Carbapenems						
Imipenem	62 (55.4%)	93 (31.3%)	2.720 (1.741-4.249)	0.000	1.326 (0.596-2.950)	0.489
Meropenem	13 (11.6%)	17 (5.7%)	2.163 (1.014-4.614)	0.046	0.864 (0.310-2.408)	0.780
Glycopeptides						
Vancomycin	57 (50.9%)	88 (29.6%)	2.461 (1.575-3.846)	0.000	0.725 (0.314-1.673)	0.451
Fluoroquinolones						
Levofloxacin	16 (14.3%)	19 (6.4%)	2.439 (1.206-4.932)	0.013	1.111 (0.435-2.837)	0.825
Oxazolidones						
Linezolid	17 (15.2%)	26 (8.8%)	1.865 (0.969-3.589)	0.062		
Sulfonamides						
Trimethoprim/sulfamethoxazole	79 (70.5%)	189 (63.6%)	1.368 (0.855-2.189)	0.191		
Tetracyclines						
Tigecycline	16 (14.3%)	26 (8.8%)	1.737 (0.894-3.377)	0.104		
Polymyxins						
Polymyxin B	12 (10.7%)	9 (3.0%)	3.840 (1.571-9.386)	0.003	0.495 (0.141-1.741)	0.273
Antifungals						
Nystatin	102 (91.1%)	290 (97.6%)	0.246 (0.091-0.664)	0.994		
Voriconazole	27 (24.1%)	67 (22.6%)	1.090 (0.654-1.818)	0.740		
Posaconazole	33 (29.5%)	23 (7.7%)	4.976 (2.763-8.962)	0.000	3.191 (1.495-6.811)	0.003
Caspofungin	16 (14.3%)	31 (10.4%)	1.430 (0.749-2.731)	0.278		
Amphotericin B	10 (8.9%)	9 (3.0%)	3.137 (1.240-7.939)	0.016	1.203 (0.344-4.214)	0.772
Antivirals						
Entecavir	77 (68.8%)	162 (54.5%)	1.833 (1.157-2.905)	0.010	1.621 (0.898-2.927)	0.109
Acyclovir	59 (52.7%)	45 (15.2%)	6.234 (3.827-10.156)	0.000	3.586 (1.908-6.739)	0.000
Oseltamivir	12 (10.7%)	24 (8.1%)	1.365 (0.658-2.832)	0.403		
Proton pump inhibitor						
Omeprazole	95 (84.8%)	170 (57.2%)	4.175 (2.373-7.345)	0.000	2.462 (1.240-4.892)	0.010

Kp, *Klebsiella pneumoniae*; HCT-CI, hematopoietic cell transplantation–comorbidity index; ECOG, Eastern Cooperative Oncology Group; AML, acute myelogenous leukemia; ALL, acute lymphoblastic leukemia; MM, multiple myeloma; LYM, lymphoma; MDS, myelodysplastic; AA, aplastic anemia; PICC, Peripherally Inserted Central Catheter. OR, odds ratio. *P* value less than 0.05.

**Table 4:** Accuracy of the proposed scoring system for the diagnosis of Kp-colonized.

Score	KP colonized (n =112)	Non-colonized (n =297)	Sensitivity	Specificity	Youden’s index (J)
≥ 0	112	297	1.00	0	0
≥ 1	111	263	0.99	0.11	0.10
≥ 2	103	199	0.92	0.33	0.25
≥ 3	93	127	0.83	0.57	0.40
≥ 4	70	76	0.63	0.74	0.37
≥ 5	60	44	0.54	0.85	0.39
≥ 6	43	25	0.38	0.92	0.30

Kp, Klebsiella pneumoniae.

In this case-control analysis (54 CTX-M-Kp colonized *vs*. 108 non-CTX-M-Kp colonized HSCT recipients), multivariate analysis identified the following independent risk factors for CTX-M-Kp colonization among HSCT recipients (*P* < 0.05): higher HCT-CI (OR 10.525, 95% CI 3.178-34.860; *P* < 0.0001), imipenem exposure (OR 3.679, 95% CI 1.047-12.929; *P* < 0.05), acyclovir administration (OR 4.403, 95% CI 1.373-14.114; *P* < 0.05) (**Supplementary Table 2**). The ROC analysis confirmed that the model possesses high discriminatory power, with an area under the curve (AUC) of 0.894 (**Figure 3B**).

In this case-control analysis (14 CRKp colonized *vs*. 28 non-CRKp colonized HSCT recipients), given the limited microbial sample size, which may introduce bias, this study employed a stratified multivariate logistic regression analysis approach. Specifically, based on univariate analysis results (*P* < 0.05), two independent multivariate models were constructed: Model 1 incorporated all significant variables except those related to medication history, while Model 2 specifically incorporated significant variables related to medication history. Multivariate analysis identified fever (OR 21.944, 95% CI 1.692-284.572; *P* < 0.05) and history of imipenem use (OR 25.574, 95% CI 1.694-386.016; *P* < 0.05) as key risk factors (**Supplementary Table 3**). Within Model 1, fever demonstrated statistical significance (*P* < 0.05). The ROC analysis confirmed that the model possesses high discriminatory power, with an AUC of 0.949 (**Figure 3C**). Similarly, within Model 2, history of imipenem use showed statistical significance (*P* < 0.05). The ROC analysis confirmed that the model possesses high discriminatory power, with an AUC of 0.920 (**Figure 3D**).

### Risk factors for subsequent BSI following colonization

This study analyzed the occurrence of bloodstream infections within 3 months following perianal swab screening in 409 HSCT patients. The results revealed that 81 patients developed subsequent bloodstream infections. Among the 81 HSCT patients with BSIs included in this study, the distribution of bacterial pathogens revealed that *Escherichia coli* was the predominant causative agent (n=22, 27.2%), followed by Kp (n=20, 24.7%). Additional isolated pathogens comprised *Enterococcus faecium* and *Stenotrophomonas maltophilia*, along with other species (**Table 5**).

**Table 5:** Microorganisms distribution of subsequent bloodstream infections in Kp-colonized versus non-colonized group.

Clinical isolates	Kp-colonization group (*n* = 112)	Non-colonization group (*n* = 297)
*Escherichia coli*	7	15
*Klebsiella pneumoniae*	14	6
*Klebsiella ozaenae*	0	1
*Enterobacter cloacae*	1	0
*Proteus mirabilis*	1	0
*Pseudomonas aeruginosa*	0	2
*Stenotrophomonas maltophilia*	1	5
*Burkholderia cepacia*	1	0
*Staphylococcus aureus*	0	2
*Staphylococcus epidermidis*	0	2
*Staphylococcus haemolyticus*	1	2
*Staphylococcus capitis*	0	1
*Streptococcus anginosus group*	1	0
*Streptococcus mitis*	0	2
*Streptococcus oralis*	0	3
*Enterococcus faecalis*	0	1
*Enterococcus faecium*	1	5
*Listeria monocytogenes*	0	1
*Candida tropicalis*	1	0
*Candida parapsilosis*	0	1
*Cryptococcus neoformans*	0	1
*Fusarium* spp.	0	1
*E. coli+ PA*	0	1

Kp, Klebsiella pneumoniae; PA, Pseudomonas aeruginosa.

Among the 112 patients with intestinal Kp colonization, 29 developed bloodstream infections (including 14 cases of Kp infection). Among the 297 patients without Kp colonization, 52 developed bloodstream infections (including 6 cases of Kp infection). To assess the impact of intestinal Kp colonization on subsequent Kp bloodstream infection in HSCT patients, an analytical model was established using intestinal Kp colonization status as the independent variable and subsequent Kp bloodstream infection as the dependent variable. Intergroup comparison demonstrated: Within the Kp bloodstream infection group (n=20), 6 patients had no history of Kp colonization while 14 were colonized with Kp. Within the non-Kp bloodstream infection group (n=389), 291 patients had no colonization history and 98 were colonized.

Logistic regression analysis confirmed that intestinal Kp colonization is an independent risk factor for subsequent Kp bloodstream infection (OR 6.929, 95% CI: 2.592-18.523; *P* < 0.0001) (**Table 6**).

**Table 6:** Logistic regression analysis of risk factors for Kp BSI development in HSCT recipients.

Variables	Kp-colonization group (*n* = 112)	Non-colonization group (*n* = 297)	OR (95%CI)	*P*-value
Secondary KP BSI	14 (12.5%)	6 (2.0%)	6.929 (2.592-18.523)	<0.0001
No secondary KP BSI	98 (87.5%)	291 (98.0%)		

Kp, *Klebsiella pneumoniae*; BSI, bloodstream infection; HSCT, hematopoietic stem cell transplantation; OR, odds ratio. *P* value less than 0.05.

## Discussion

Currently, healthcare institutions lack systematic monitoring of Kp colonization in HSCT recipients, resulting in a continuous increase in the risk of Kp transmission. Studies have shown that screening for asymptomatic colonization in patients and implementing contact precautions can effectively reduce the risk of transmission between patients (Schneider et al., 2019). Furthermore, an in-depth investigation into the prevalence of Kp colonization among patients in HSCT wards is a core step in formulating precise infection control strategies and establishing protective barriers for vulnerable populations. This study achieves two key innovative breakthroughs: it is the first to systematically explore the characteristics of drug resistance gene carriage in HSCT recipients, and simultaneously the first to establish a correlation model between medication use in HSCT recipients and intestinal Kp colonization. The research data indicate that the risk of intestinal Kp colonization is significantly increased in patient groups using drugs such as posaconazole, omeprazole, and acyclovir. These findings provide multi-dimensional scientific evidence for the upgrading of infection prevention and control systems in HSCT wards: from an etiological perspective, they emphasize the necessity of establishing a systematic Kp colonization monitoring network; from the perspective of clinical medication, they offer data support for optimizing drug selection strategies. It is expected to promote the formation of a closed-loop prevention and control system integrating “drug resistance gene monitoring, precise medication management, and colonization prevention and control”.

The intestinal tract serves as an important reservoir for Kp (Le Bris et al., 2025). The rate of intestinal colonization by Kp varies significantly among different populations and exhibits distinct population specificity and geographical characteristics (Giannella et al., 2014). In the foreign community populations, the intestinal Kp colonization rate in healthy individuals fluctuates widely (16%-90%) (Budel et al., 2019; Lin et al., 2012; Raffelsberger et al., 2023; Ahmed et al., 2014). In the Chinese community populations, survey data from 2015 to 2017 showed a relatively low intestinal Kp colonization rate, ranging from 12% to 27.5% (Wang et al., 2022). Compared with community populations, inpatients, especially various high-risk groups, have significantly higher intestinal Kp colonization rates: 54.9% in neonates (Moussa et al., 2023), and 28% in Intensive Care Unit (ICU) patients (Qin et al., 2020). In this study, the intestinal Kp colonization rate in HSCT patients was 28.6%, which is not only higher than that in Chinese community populations but also close to the reported data in the abovementioned high-risk inpatients (e.g., ICU patients). This preliminarily suggests that HSCT patients are a high-risk group for intestinal Kp colonization.

The emergence of extended-spectrum β-lactamase-producing Kp (ESBL-Kp) poses a growing global public health challenge due to its resistance to critical antibiotics including broad-spectrum cephalosporins. This study reveals an alarming finding: the intestinal colonization rate of CTX-M-type ESBL-producing Kp (CTX-M-Kp) in the study population reached 48.0%. This figure substantially exceeds the average ESBL-Kp colonization rates (typically ranging from 12.1% to 24.5%) reported in most studies of general hospitalized patients (Denkel et al., 2020; Kizilates et al., 2021; Aghamohammad et al., 2020; Kiddee et al., 2019), and approaches the levels previously reported in Neonatal Intensive Care Unit (NICUs) (54.8%) (Moussa et al., 2023). Given that ESBL-Kp (particularly CTX-M variants) serves as an important pathogen for nosocomial infections, with intestinal colonization being a critical risk factor for subsequent infections (Giannella et al., 2014), the abnormally high colonization rate of CTX-M-Kp observed in this study demands urgent attention.

In recent years, the prevalence of CRKp has risen rapidly in China (Giannella et al., 2014). Notably, this study identified a CRKp intestinal colonization rate of merely 3.4% in HSCT patients—a finding consistent with reports in patients with hematologic malignancies (Chen et al., 2023; Zhu et al., 2022). Consistent with our previous observation of rare carbapenemase genes detection in this population (only *bla_KPC_* (1.8%) and *bla_NDM_* (3.6%) genes were identified). This finding confirms the low prevalence of carbapenemase-resistant genes in gut-colonized Kp among HSCT recipients. The current genetic evidence further supports reduced intestinal carriage and transmission potential of carbapenem resistance in the gut microbiota of HSCT patients.

This study revealed high carriage rates of resistance gene types: All strains carried ESBL resistance genes (100%), with a 97.3% detection rate for quinolone resistance genes, while aminoglycoside resistance genes were identified in 85.7% of the isolates. Given the current paucity of systematic research data in this field, whether the specific resistance pattern observed in the gut microbiota of HSCT patients—characterized by a low burden of carbapenemase resistance genes alongside high carriage of ESBL, quinolone and aminoglycoside resistance genes—represents a prevalent phenomenon, and how it potentially differs from other populations, warrants further validation through large-scale, multi-center studies.

The human intestinal tract constitutes a complex and dynamically evolving microecosystem (Unverdorben et al., 2025). The implantation and sustained colonization of Kp within this environment involve competitive interactions with commensal gut microbiota and adaptation to diverse environmental stressors—a process finely regulated by multiple factors (Cai et al., 2024). In addition, previous studies of this research have proven that the colonization rate of Kp is relatively high in HSCT patients (28.6%). Consequently, elucidating the risk factors for Kp colonization in HSCT patients, is crucial for developing effective strategies to control Kp colonization.

This study identified HCT-CI, fever, posaconazole, acyclovir, and omeprazole as independent risk factors for intestinal colonization of Kp in HSCT patients. Among these, the HCT-CI score is used to quantitatively assess the specific mortality risk in transplant patients, with those having higher scores often complicated by diabetes, liver dysfunction, or other malignant tumors (Pan et al., 2025). These comorbidities, on one hand, impair the patients’ immune function and, on the other hand, disrupt the integrity of the intestinal mucosal barrier, thereby creating favorable conditions for Kp colonization (Pan et al., 2025).

Omeprazole, a type of proton pump inhibitor (PPI), can reduce gastric acid secretion, alter the intestinal acid-base environment, and further disrupt the homeostasis of intestinal microbiota (Tuominen et al., 2025). Existing studies have pointed out that individuals with intestinal diseases such as Crohn’s disease and ulcerative colitis, as well as those using PPIs, have a significantly increased risk of intestinal bacterial colonization (Raffelsberger et al., 2021). In HSCT patients, PPI treatment exacerbates immunosuppression and imbalances the intestinal ecological environment, making them more susceptible to intestinal Kp colonization and subsequent infections (Imhann et al., 2016; Safiri and Sullman, 2018; Cunningham et al., 2018).

Posaconazole, a second-generation triazole antifungal agent (Yawalkar et al., 2025), and acyclovir, an antiviral nucleoside analog (Sun et al., 2025), are commonly used in HSCT patients for antifungal and antiviral therapy, respectively. However, there are currently no reports on the impact of these two drugs on intestinal Kp colonization. Given the severely compromised immune function of HSCT patients, extensive use of posaconazole and acyclovir may excessively inhibit normal intestinal flora growth, disrupt intestinal microecological balance, induce intestinal microcirculatory disorders, and ultimately lead to Kp colonization. Therefore, to reduce Kp colonization in HSCT patients, the use of these two drugs should be utilized appropriately.

Among the factors influencing the colonization of CTX-M-Kp in HSCT patients, HCT-CI, use of imipenem, and acyclovir were identified as independent risk factors. For the colonization of CRKp in HSCT patients, prior use of imipenem was an independent risk factor, and imipenem was also a risk factor for CTX-M-Kp colonization. As a carbapenem antibiotic, imipenem is widely used in the treatment of infections caused by Gram-negative bacilli. However, its irrational and excessive use has led to persistently high detection rates of resistant strains (Tzouvelekis et al., 2014; Agyeman et al., 2020). Studies related to ICU patients have indicated that exposure to carbapenems is a risk factor for intestinal colonization by carbapenem-resistant Enterobacteriaceae (CRE) (Yuan et al., 2022); another study involving ICU and hematological patients showed that the probability of intestinal CRE colonization in patients exposed to carbapenems was 2.2 times higher than that in non-exposed patients (Chen et al., 2022). These findings are consistent with the results of the present study, fully demonstrating that the use of carbapenems significantly increases the risk of intestinal CRKp colonization in patients. In addition, fever and the use of imipenem are also independent risk factors for CRKp colonization in HSCT patients. Fever can cause immune dysfunction in patients, and the use of imipenem further disrupts the intestinal microecology. The synergistic effect of these two factors promotes the intestinal colonization of CRKp.

Intestinal colonization by Kp constitutes a critical risk factor for secondary infections in hospitalized patients, particularly BSI (Raffelsberger et al., 2021; Sun et al., 2021). As early as 1971, Selden et al. first proposed, through studies on capsular serotyping of Kp, that gastrointestinal colonization might serve as a significant reservoir for strains causing healthcare-associated infections, including BSI, albeit with the limitation that serotyping is insufficient to differentiate closely related strains (Selden et al., 1971). A 1990 study further revealed that the serotypes’ similarity between clinically isolated strains and fecal isolates was significantly higher than that between clinical isolates and strains from natural environments, thereby providing more robust evidence for the association between colonization and infection (Podschun, 1990). A 2012 prospective study involving 497 hematological patients explicitly confirmed that prior intestinal colonization represents the primary risk factor for secondary BSI (Vehreschild et al., 2014).

This study observed that *Escherichia coli* and Kp are the most predominant bacterial species in BSI among HSCT patients. This phenomenon is closely related to their biological characteristics and adaptability to clinical environments: first, both are common members of the normal human intestinal flora, endowed with an inherent advantage for long-term intestinal colonization; second, they can acquire various virulence factors (such as adhesins and hemolysins in *E.coli*, and capsular polysaccharides in Kp) to enhance their adhesion and invasion capabilities, thereby breaking through the mucosal barrier; third, both bacteria are prone to acquiring resistance genes via mobile genetic elements such as plasmids and transposons, enabling them to survive and overproliferated more easily in medical environments with extensive use of broad-spectrum antibiotics (Fu et al., 2024); furthermore, due to factors such as immunosuppression, intestinal microecological imbalance, and mucosal damage in HSCT patients, their ability to clear these two opportunistic pathogens is significantly reduced, further facilitating their dominance in BSI.

Meanwhile, our risk assessment in HSCT patients revealed that those with intestinal Kp colonization faced a substantially elevated risk of subsequent Kp BSI compared to their non-colonized counterparts. This finding demonstrates remarkable consistency with the hazard ratio of 6.062 reported in acute leukemia patients (Zhu et al., 2022), robustly confirming the strong association between intestinal Kp colonization and subsequent BSI. The underlying mechanism stems from the patient’s endogenous gut microbiota serving as the primary reservoir for infecting strains. Even when present at low intestinal burdens, these strains can precipitate infection under permissive conditions (Martin et al., 2016; Gorrie et al., 2017; Amaretti et al., 2020; Rao et al., 2021). In HSCT recipients specifically, chemotherapy and transplant-related treatments induce severe immunosuppression, disrupt intestinal mucosal barriers, and cause ecological dysbiosis—collectively heightening the risk of Kp translocation from the gut into the bloodstream and subsequent BSI (Almasian Tehrani et al., 2023). Furthermore, experimental evidence supports this clinical association: Joseph et al. established a murine model recapitulating the progression from gastrointestinal Kp colonization to systemic infection (including urinary tract, pulmonary, and bloodstream infections), elucidating bacterial functional determinants mediating colonization-to-dissemination transition (Joseph et al., 2021). Complementarily, investigators from China have utilized alternative non-mammalian models (zebrafish and Galleria mellonella) to confirm both the feasibility of infection following intestinal colonization and host-specific immunological variations in susceptibility (Zhang et al., 2019). Collectively, these experimental models provide mechanistic validation for the clinical link between Kp intestinal colonization and BSI.

Mitigating Kp colonization risk necessitates a bundled strategy at the antimicrobial stewardship and infection control interface. First, tailoring empirical antibiotic regimens based on local Kp epidemiology and de-escalating therapy once culture results are available can reduce selection pressure. Furthermore, the rational management of commonly used supportive medications, based on our findings, is crucial. Judicious use of drugs such as omeprazole, posaconazole, and acyclovir—which were identified as potential modifiable risk factors for Kp colonization—may effectively reduce the intestinal colonization burden in HSCT patients. Second, reinforcing environmental hygiene (given Kp’s ability to persist on surfaces) and ensuring strict hand hygiene compliance are fundamental, low-tech yet highly effective measures to break transmission chains.

## Conclusions

This study is the first to systematically characterize the antimicrobial resistance profile of intestinal colonizing Kp in HSCT patients, revealing a unique pattern characterized by a low burden of carbapenem resistance genes yet high carriage rates of ESBL, quinolone, and aminoglycoside resistance determinants. These findings provide critical experimental evidence for understanding resistance gene transmission dynamics and inform precise infection prevention strategies in this vulnerable population. Given the profound immunosuppression of HSCT recipients and the plasmid-mediated transferability of highly prevalent resistance genes such as ESBLs, routine surveillance of intestinal Kp colonization is of substantial clinical importance. Furthermore, our data suggest that judicious management of non-antibiotic agents—specifically omeprazole, posaconazole, and acyclovir—may represent a feasible adjunctive strategy to reduce Kp colonization risk, interrupt progression to bloodstream infection, and ultimately improve patient outcomes.

## Data Availability

The original contributions presented in the study are included in the article/**Supplementary Material**. Further inquiries can be directed to the corresponding authors.
