# Correction: Characteristics of the resistome and the potential for bloodstream infections in patients with gut colonization by *Klebsiella pneumoniae* undergoing hematopoietic stem cell transplantation

**DOI:** 10.3389/fcimb.2026.1856712

**Published:** 2026-04-27

**Authors:** Jiacheng Qiu, Zhenxin Zhuang, Rongping Zhu, Yingping Cao, Xiaohong Xu

**Affiliations:** 1 Department of Laboratory Medicine, Fujian Medical University Union Hospital, Fuzhou, Fujian, China; 2 Department of Laboratory Medicine, Jinjiang Hospital of Traditional Chinese Medicine Affiliated to Fujian University of Traditional Chinese Medicine, Quanzhou, Fujian, China; 3 Department of Blood Transfusion, Fuzhou University Affiliated Provincial Hospital, Fuzhou, Fujian, China; 4 Fujian Medical University Union Clinical College, Fuzhou, Fujian, China

**Keywords:** bloodstream infections, colonization, hematopoietic stem cell transplantation, *Klebsiella pneumoniae*, non-antibacterial drugs, risk factors

Affiliation “1. Department of Laboratory Medicine, Fujian Medical University Union Hospital, Fuzhou, Fujian, China

2. Department of Laboratory Medicine, Jinjiang Hospital of Traditional Chinese Medicine Affiliated to Fujian University of Traditional Chinese Medicine, Quanzhou, Fujian, China

3. Department of Blood Transfusion, Fuzhou University Affiliated Provincial Hospital, Fuzhou, Fujian, China

4. Fujian Medical University Union Clinical College, Fuzhou, Fujian, China” was erroneously given as “1. Department of Laboratory Medicine, Fujian Medical University Union Hospital, Fuzhou, Fujian, China, 2. Department of Laboratory Medicine, Jinjiang Hospital of Traditional Chinese Medicine Affiliated to Fujian University of Traditional Chinese Medicine, Quanzhou, Fujian, China,

3. Department of Laboratory Medicine, Fuqing City Hospital Affiliated to Fujian Medical University, Fuqing, Fujian, China,

4. Department of Blood Transfusion, Fuzhou University Affiliated Provincial Hospital, Fuzhou, Fujian, China,

5. Fujian Medical University Union Clinical College, Fuzhou, Fujian, China”.

The original version of this article has been updated.

