# Supplementary material for: Characteristics of the resistome and the potential for bloodstream infections in patients with gut colonization by Klebsiella pneumoniae undergoing hematopoietic stem cell transplantation
Source: Front Cell Infect Microbiol. 2026 Mar 27;16:1727300. doi: 10.3389/fcimb.2026.1727300 (PMC13066209; doi:10.3389/fcimb.2026.1727300)
Supplement: Supplementary file 1 [file Table1.docx]

**Table S1** Primer sequences used in polymerase chain reaction for resistance genes detection

| Primer name | Sequence (5’-3’) | Product size |
| --- | --- | --- |
| *SHV*-F | TTATCTCCCTGTTAGCCACC | 779 bp |
| *SHV*-R | GATTTGCTGATTTCGCTCGG |  |
| *TEM*-F | ATAAAATTCTTGAAGACGAAA | 650 bp |
| *TEM* -R | GACAGTTACCAATGCTTAATC |  |
| *CTX-M-1*-F | GCATTGATTAACACAGCAGATA | 650 bp |
| *CTX-M-1*-R | GTGAAGTAAGTGACCAGAATC |  |
| *CTX-M-2*-F | ATAATTCGCAGATTCTCTACC | 631 bp |
| *CTX-M-2*-R | GTAAAGTAGGTCACCAGAAC |  |
| *CTX-M-8*-F | AAGAAGGTGTTGAGTCAGAA | 471 bp |
| *CTX-M-8*-R | GTCATTCGTCGTACCATAATC |  |
| *CTX-M-9*-F | ATGGTGACAAAGAGAGTGCA | 870 bp |
| *CTX-M-9*-R | CCCTTCGGCGATGATTCTC |  |
| *CTX-M-10*-F | GCAGCACCAGTAAAGTGATGG | 524 bp |
| *CTX-M-10*-R | GCGATATCGTTGGTGGTACC |  |
| *CTX-M-14*-F | GAGAGTGCAACGGATGATG | 941 bp |
| *CTX-M-14*-R | TGCGGCTGGTAAAATAG |  |
| *qnrA*-F | AGAGGATTTCTCACGCCAGG | 580 bp |
| *qnrA*-R | TGCCAGGCACAGATCTTGAC |  |
| *qnrB*-F | GGMATHGAAATTCGCCACTG | 264 bp |
| *qnrB*-R | TTTGCYGYYCGCCAGTCGAA |  |
| *qnrS*-F | GCAAGTTCATTGAACAGGGT | 428 bp |
| *qnrS*-R | TCTAAACCGTCGAGTTCGGCG |  |
| *qepA*-F | AGGTACTGCGTCATGAAGAT | 716 bp |
| *qepA*-R | CTGGACATCTACGGCTTCTT |  |
| *gyrA*-F | CTTATCTGGATTATGCGATGTC | 515 bp |
| *gyrA* -R | AATCACTTCCGTCAGGTTAT |  |
| *aac(6’)-Ib-*F | TTGCGATGCTCTATGAGTGGCTA | 482 bp |
| *aac(6’)-Ib-*R | CTCGAATGCCTGGCGTGTTT |  |
| *aac(3)-Ⅱ*-F | TGAAACGCTGACGGAGCCTC | 370 bp |
| *aac(3)-Ⅱ*-R | GTCGAACAGGTAGCACTGAG |  |
| *ant (3’’)-I-F* | GTGGATGGCGGCCTGAAGCC | 526 bp |
| *ant (3’’)-I -R* | ATTGCCCAGTCGGCAGCG |  |
| *armA*-F | ATACTAACTCATTCCCTATAACCT | 589 bp |
| *armA*-R | TCTTACTATTCTGCCTATCCTAAT |  |
| *rmtB*-F | CTGGATACCCTGTACGATTT | 461 bp |
| *rmtB*-R | ATAAGTTCTGTTCCGATGGT |  |
| *bla_KPC_*-F | GTGTTTCCCTTTAGCCAATC | 622 bp |
| *bla_KPC_*-R | TAGTTCTGCTGTCTTGTCTC |  |
| *bla_IMP_-F* | ATGCTGAGGCTTATCTAATTG | 430 bp |
| *bla_IMP_-R* | TTTAATAATTTGGCGGACTTTG |  |
| *bla_NDM_*-F | CACCTCATGTTTGAATTCGCC | 951 bp |
| *bla_NDM_*-R | CTCTGTCACATCGAAATCGC |  |
| *bla_VIM_* -F | AGTAGTTTATTGGTCTACATGAC | 550 bp |
| *bla_VIM_*-R | GGACGTATACAACCAGATTG |  |
| *bla_OXA-48_*-F | TTGGTGGCATCGATTATCGG | 744 bp |
| *bla_OXA-48_*-R | GAGCACTTCTTTTGTGATGGC |  |
| *bla_OXA-181_*-F | AGTTGTGCCTGTTTATCAAG | 306 bp |
| *bla_OXA-181_*-R | CCAATCTTAGGTTCGATTCTAG |  |
| *IMI*-F | TGCGGTCGATTGGAGATAAA | 399 bp |
| *IMI*-R | CGATTCTTGAAGCTTCTGCG |  |
| *GIM*-F | CGAATGGGTTGGTAGTTCTGGATAATAATC | 198 bp |
| *GIM-*R | ATGTGTATGTAGGAATTGACTTTGAATTTA |  |
| *SME-*F | AACGGCTTCATTTTTGTTTAG | 831 bp |
| *SME*-R | GCTTCCGCAATAGTTTTATCA |  |

**Table S2** Univariate analysis and multivariate logistic regression analysis of risk factors for CTX-M-Kp colonization in colonized versus non-colonized patients

| **Variables** | **CTX-M-Kp colonized n (%)** | **Non-colonized n (%)** | **Bivariate** | | **Multivariate** | |
| --- | --- | --- | --- | --- | --- | --- |
| **Total no. of patients** | **N=54 (33.3)** | **N=108(66.7)** | **OR (95%CI)** | **p** | **OR (95%CI)** | **p** |
| **Age (years)** | 39.4±16.1 | 39.2±18.4 | 1.001 (0.982-1.019) | 0.952 |  |  |
| **Sex (male)** | 32 (59.3%) | 58 (53.7%) | 1.254 (0.647-2.430) | 0.503 |  |  |
| **HCT-CI (≥3)** | 27 (50.0%) | 9 (8.3%) | 11.000 (4.626-26.155) | 0.000 | 10.525 (3.178-34.860) | 0.000 |
| **ECOG (3 to 5)** | 9 (16.7%) | 7 (6.5%) | 2.886 (1.012-8.233) | 0.048 | 2.318 (0.468-11.488) | 0.303 |
| **Hospital stays (>30 days)** | 36 (66.7%) | 41 (38.0%) | 3.268 (1.645-6.493) | 0.001 | 2.826 (0.944-8.460) | 0.063 |
| **No. of admissions (>5 times)** | 11 (20.4%) | 32 (29.6%) | 0.608 (0.278-1.326) | 0.211 |  |  |
| **Underlying Disease** | **n (%)** | **n (%)** | **OR (95% CI)** | **p** | **OR (95% CI)** | **p** |
| AML | 16 (29.6%) | 21 (19.4%) | 0.657 (0.226-1.912) | 0.441 |  |  |
| ALL | 20 (37.0%) | 24 (22.2%) | 2.424 (0.979-6.000) | 0.055 |  |  |
| MM | 7 (13.0%) | 31 (28.7%) | 2.216 (0.862-5.700) | 0.099 |  |  |
| Others | 11 (20.4%) | 32 (29.6%) |  |  |  |  |
| **Comorbidity** |  |  |  |  |  |  |
| Hypertension | 4 (7.4%) | 9 (8.3%) | 0.880 (0.258-2.998) | 0.838 |  |  |
| Diabetes mellitus | 3 (5.6%) | 6 (5.6%) | 1.000 (0.240-4.162) | 1.000 |  |  |
| Solid tumor | 1 (1.9%) | 7 (6.5%) | 0.272 (0.033-2.271) | 0.229 |  |  |
| Cardiac disease | 5 (9.3%) | 8 (7.4%) | 1.276 (0.396-4.103) | 0.683 |  |  |
| Respiratory disease | 14 (25.9%) | 21 (19.4%) | 1.450 (0.669-3.141) | 0.346 |  |  |
| Gastrointestinal disease | 5 (9.3%) | 5 (4.6%) | 2.102 (0.581-7.601) | 0.257 |  |  |
| Hepatic disease | 11 (20.4%) | 21 (19.4%) | 1.060 (0.469-2.396) | 0.889 |  |  |
| Urinary system disease | 6 (11.1%) | 18 (16.7%) | 0.625 (0.233-1.679) | 0.351 |  |  |
| **Invasive operation** | **n (%)** | **n (%)** | **OR (95% CI)** | **p** | **OR (95% CI)** | **p** |
| Past surgical history | 15 (27.8%) | 25 (23.1%) | 1.277 (0.606-2.689) | 0.520 |  |  |
| **Fever** | 25 (46.3%) | 21 (19.4%) | 3.571 (1.745-7.311) | 0.000 | 2.461 (0.862-7.032) | 0.093 |
| **History of antibiotic intake within last 3 months** | | | | | | |
| **β-lactam/ inhibitor** |  |  |  |  |  |  |
| Cefoperazone/sulbactam | 24 (44.4%) | 28 (25.9%) | 2.286 (1.149-4.548) | 0.019 | 0.543 (0.174-1.693) | 0.292 |
| **Cephalosporins** |  |  |  |  |  |  |
| Cefdinir | 19 (35.2%) | 14 (13.0%) | 3.645 (1.651-8.047) | 0.001 | 2.871 (0.888-9.287) | 0.078 |
| **Aminoglycosides** |  |  |  |  |  |  |
| Gentamicin | 47 (87.0%) | 108 (100.0%) | 0 | >0.999 |  |  |
| **Carbapenems** |  |  |  |  |  |  |
| Imipenem | 32 (59.3%) | 20 (18.5%) | 6.400 (3.090-13.258) | 0.000 | 3.679 (1.047-12.929) | 0.042 |
| **Glycopeptides** |  |  |  |  |  |  |
| Vancomycin | 28 (51.9%) | 23 (21.3%) | 3.980 (1.966-8.056) | 0.000 | 0.735 (0.197-2.741) | 0.647 |
| **Sulfonamides** |  |  |  |  |  |  |
| Trimethoprim/sulfamethoxazole | 42 (77.8%) | 64 (59.3%) | 2.406 (1.139-5.082) | 0.021 | 1.093 (0.358-3.341) | 0.876 |
| **Antifungals** |  |  |  |  |  |  |
| Nystatin | 51 (94.4%) | 107 (99.1%) | 0.159 (0.016-1.565) | 0.115 |  |  |
| Voriconazole | 14 (25.9%) | 13 (12.0%) | 2.558 (1.104-5.927) | 0.029 | 0.974 (0.263-3.599) | 0.968 |
| **Antivirals** |  |  |  |  |  |  |
| Entecavir | 40 (74.1%) | 56 (51.9%) | 2.653 (1.296-5.430) | 0.008 | 1.223 (0.425-3.519) | 0.709 |
| Acyclovir | 32 (59.3%) | 18 (16.7%) | 7.273 (3.463-15.276) | 0.000 | 4.403 (1.373-14.114) | 0.013 |
| **Proton pump inhibitor** |  |  |  |  |  |  |
| Omeprazole | 47 (87.0%) | 57 (52.8%) | 6.008 (2.493-14.474) | 0.000 | 2.822 (0.875-9.095) | 0.082 |

Kp, *Klebsiella pneumoniae*; HCT-CI, hematopoietic cell transplantation–comorbidity index; ECOG, Eastern Cooperative Oncology Group; AML, acute myelogenous leukemia; ALL, acute lymphoblastic leukemia; MM, multiple myeloma.

*P* value less than 0.05.

**Table S3** Univariate analysis and multivariate logistic regression analysis of risk factors for CRKp colonization in colonized versus non-colonized patients

| **Variables** | **CRKp colonized n (%)** | **Non-colonized n (%)** | **Bivariate** | | **Multivariate** | |
| --- | --- | --- | --- | --- | --- | --- |
| **Total no. of patients** | **N=14 (33.3)** | **N=28(66.7)** | **OR (95%CI)** | **p** | **OR (95%CI)** | **p** |
| **Age range (16 to 76years)** | 12 (85.7%) | 23 (82.1%) | 1.304 (0.219-7.751) | 0.770 |  |  |
| **Sex (male)** | 9 (64.3%) | 19 (67.9%) | 1.173 (0.304-4.527) | 0.817 |  |  |
| **HCT-CI (≥3)** | 12 (85.7%) | 2 (7.7%) | 72.000 (9.005-575.663) | 0.000 | 17.202 (0.859-344.308) | 0.063 |
| **ECOG (3 to 5)** | 5 (35.7%) | 2 (7.1%) | 7.222 (1.186-43.979) | 0.032 | 0 | 0.998 |
| **Hospital stays (>30 days)** | 12 (85.7%) | 14 (50.0%) | 6.000 (1.129-31.880) | 0.036 | 0 | 0.998 |
| **No. of admissions (>5 times)** | 3 (21.4%) | 11 (39.3%) | 0.421 (0.095-1.861) | 0.254 |  |  |
| **Underlying Disease** | **n (%)** | **n (%)** | **OR (95% CI)** | **p** | **OR (95% CI)** | **p** |
| AML | 3 (21.4%) | 9 (32.1%) | 1.333 (0.176-10.120) | 0.781 |  |  |
| ALL | 3 (21.4%) | 4 (14.3%) | 3.000 (0.348-25.870) | 0.318 |  |  |
| MDS | 4 (28.6%) | 2 (7.1%) | 8.000 (0.803-79.655) | 0.076 |  |  |
| MM | 2 (14.3%) | 5 (17.9%) | 1.600 (0.168-15.273) | 0.683 |  |  |
| Others | 2 (14.3%) | 8 (28.6%) |  |  |  |  |
| **Comorbidity** |  |  |  |  |  |  |
| Cardiac disease | 2 (14.3%) | 2 (7.1%) | 2.167 (0.272-17.272) | 0.465 |  |  |
| Respiratory disease | 5 (35.7%) | 5 (17.9%) | 2.556 (0.594-11.000) | 0.208 |  |  |
| Hepatic disease | 3 (21.4%) | 8 (28.6%) | 0.682 (0.150-3.109) | 0.621 |  |  |
| Urinary system disease | 2 (14.3%) | 6 (21.4%) | 0.611 (0.106-3.510) | 0.581 |  |  |
| **Invasive operation** | **n (%)** | **n (%)** | **OR (95% CI)** | **p** | **OR (95% CI)** | **p** |
| Past surgical history | 2 (14.3%) | 3 (10.7%) | 1.389 (0.204-9.445) | 0.737 |  |  |
| PICC | 12 (85.7%) | 27 (96.4%) | 0.222(0.18-2.693) | 0.237 |  |  |
| **Fever** | 12 (85.7%) | 5 (17.9%) | 27.600 (4.644-164.021) | 0.000 | 21.944 (1.692-284.572) | 0.018 |
| **History of antibiotic intake within last 3 months** | | | | | | |
| **β-lactam/ inhibitor** |  |  |  |  |  |  |
| Piperacillin/tazobactam | 5 (35.7%) | 3 (10.7%) | 4.630 (0.915-23.429) | 0.064 |  |  |
| Cefoperazone/sulbactam | 8 (57.1%) | 7 (25.0%) | 4.000 (1.026-15.599) | 0.046 | 0.279 (0.011-7.416) | 0.446 |
| **Cephalosporins** |  |  |  |  |  |  |
| Cefdinir | 3 (21.4%) | 2 (7.1%) | 3.545 (0.518-24.258) | 0.197 |  |  |
| Ceftazidime | 2 (14.3%) | 2 (7.1%) | 2.167 (0.272-17.272) | 0.465 |  |  |
| **Aminoglycosides** |  |  |  |  |  |  |
| Gentamicin | 12 (85.7%) | 28 (100.0%) | 0 | >0.999 |  |  |
| **Carbapenems** |  |  |  |  |  |  |
| Imipenem | 11 (78.6%) | 2 (7.1%) | 47.667 (6.967-326.131) | 0.000 | 25.574 (1.694-386.016) | 0.019 |
| **Glycopeptides** |  |  |  |  |  |  |
| Vancomycin | 9 (64.3%) | 2 (7.1%) | 23.400 (3.843-142.491) | 0.001 | 28.033 (0.591-1330.776) | 0.091 |
| **Sulfonamides** |  |  |  |  |  |  |
| Trimethoprim/sulfamethoxazole | 11 (78.6%) | 19 (67.9%) | 1.737 (0.386-7.807) | 0.472 |  |  |
| **Antifungals** |  |  |  |  |  |  |
| Nystatin | 12 (85.7%) | 28 (100.0%) | 0 | >0.999 |  |  |
| Voriconazole | 7 (50.0%) | 3 (10.7%) | 8.333 (1.697-40.911) | 0.009 | 1.615 (0.099-26.363) | 0.736 |
| Posaconazole | 5 (35.7%) | 4 (14.3%) | 3.333 (0.728-15.267) | 0.121 |  |  |
| Caspofungin | 2 (14.3%) | 3 (10.7%) | 1.389 (0.204-9.445) | 0.737 |  |  |
| **Antivirals** |  |  |  |  |  |  |
| Entecavir | 9 (64.3%) | 14 (50.0%) | 1.800 (0.481-6.742) | 0.383 |  |  |
| Acyclovir | 7 (50.0%) | 3 (10.7%) | 8.333 (1.697-40.911) | 0.009 | 5.784 (0.308-108.578) | 0.241 |
| **Proton pump inhibitor** |  |  |  |  |  |  |
| Omeprazole | 12 (85.7%) | 14 (50.0%) | 6.000 (1.129-31.880) | 0.036 | 1.153 (0.114-11.672) | 0.904 |

CRKp, carbapenem-resistant *Klebsiella pneumoniae*; HCT-CI, hematopoietic cell transplantation–comorbidity index; ECOG, Eastern Cooperative Oncology Group; AML, acute myelogenous leukemia; ALL, acute lymphoblastic leukemia; MDS, myelodysplastic; MM, multiple myeloma; PICC, Peripherally Inserted Central Catheter.

*P* value less than 0.05.
